# Test Assembly for Cognitive Diagnosis Using Mixed-Integer Linear Programming

**DOI:** 10.3389/fpsyg.2021.623077

**Published:** 2021-02-02

**Authors:** Wenyi Wang, Juanjuan Zheng, Lihong Song, Yukun Tu, Peng Gao

**Affiliations:** ^1^School of Computer and Information Engineering, Jiangxi Normal University, Nanchang, China; ^2^School of Education, Jiangxi Normal University, Nanchang, China

**Keywords:** cognitive diagnosis, cognitive diagnostic model information index, cluster analysis, mixed-integer linear programming, inter-class distance, correct classification rate

## Abstract

One purpose of cognitive diagnostic model (CDM) is designed to make inferences about unobserved latent classes based on observed item responses. A heuristic for test construction based on the CDM information index (CDI) proposed by Henson and Douglas (2005) has a far-reaching impact, but there are still many shortcomings. He and other researchers had also proposed new methods to improve or overcome the inherent shortcomings of the CDI test assembly method. In this study, one test assembly method of maximizing the minimum inter-class distance is proposed by using mixed-integer linear programming, which aims to overcome the shortcomings that the CDI method is limited to summarize the discriminating power of each item into a single CDI index while neglecting the discriminating power for each pair of latent classes. The simulation results show that compared with the CDI test assembly and random test assembly, the new test assembly method performs well and has the highest accuracy rate in terms of pattern and attributes correct classification rates. Although the accuracy rate of the new method is not very high under item constraints, it is still higher than the CDI test assembly with the same constraints.

## Introduction

The theory of cognitive diagnostic assessment (CDA) is an important part of personalized adaptive learning (Sia and Lim, 2018). Since the cognitive diagnostic model (CDM) was put forward, it has attracted much attention because of its ability to analyze and explain the test results in detail (Hsu et al., 2020). On the other hand, the test is the bridge between the abstract and unobservable ability of the examinees and the real observable item response data, so the quality of the test affects the quality of diagnostic classification directly. A test that meets the test specification needs to be selected from an item bank, then the test assembly will be restricted by many conditions and requirements (Zijlmans et al., 2019; Tang and Zhan, 2020), such as the difficulty and discrimination under the constraints of psychometrics, the maximum number of knowledge points allowed in a test, or the requirements of parallel tests.

How to construct a test with higher quality has always been a research hotspot. In the aspect of test assembly based on cognitive diagnosis, the test assembly method of CDM information index (CDI) proposed by Henson and Douglas (2005) is of great influence. Henson et al. (2008) put forward the attribute level discrimination index (ADI) under uniform and non-uniform distribution of attributes. However, neither the CDI method nor the ADI method considers the attribute hierarchical structure. When these methods are applied in practice, the performance of CDI and ADI methods will be poor under some conditions if the hierarchical structure exists between attributes (Kuo et al., 2016). In addition, Finkelman et al. (2009) proposed a method of test assembly based on genetic algorithm minimizing the expected posterior error rate for attributes under the framework of CDA. For example, the method of test assembly based on genetic algorithm takes three fitness functions: the average number of classification errors, maximum error rate, and ability to hit attribute-level target error rates. This method can directly optimize the classification errors, but its computational intensity is considerably greater than that of analytic procedures like the CDI. For classroom or formative assessment, we should choose the algorithm with low computational complexity if other algorithms for test assembly are sufficient to meet the needs (Clark, 2013).

In terms of test assembly methods based on cognitive diagnosis, researchers have proposed a large number of methods, but most of these methods are based on a certain CDI, and there are some problems such as lacking of global consideration or requiring large amount of computation. Therefore, it is urgent to consider the global information and the method of test assembly with less calculation in cognitive diagnosis. The method of test assembly based on CDI takes into account the sum of the whole amount of information, but it has been found that this method is not the optimal method of test assembly. In some cases, the total amount of information is the largest, which may due to some of the larger information on a non-trivial subset of the universal set of latent classes (i.e., the set of all possible combinations of attributes). The discriminating power of this strategy with the largest CDI is not necessarily better than the strategy with uniform distribution of information and less overall information. Therefore, the goal of this study is to explore a new method for test construction, and combine the idea of cluster analysis (Guo et al., 2020) and mixed-integer linear programming method (Kantor et al., 2020) to propose a method to maximize the minimum distance (MMD) between latent classes, in order to overcome the shortcomings of the existing methods.

## Methods

### Cognitive Diagnostic Model

The purpose of cognitive diagnostic model is to describe the relationship between examinee’s item response and his or her potential cognitive attributes (Mao, 2014). It is a psychometric model. The common cognitive diagnostic models are the deterministic input noisy “and” gate (DINA) model, the deterministic input noise “or” gate (DINO) model, and the reduced-reparameterized unified model (R-RUM; Hartz, 2002). The new method proposed in this study mainly focused on these two cognitive diagnosis models. Let K be the number of attributes to be measured by the test. The entry *q*_*jk*_ in the Q-matrix indicates whether the attribute *k* is measured in item j. When *q*_*jk*_ = 1, the attribute *k* is measured by item *j*. And 0 indicates that it has not been measured. α_*ik*_ indicates the attribute status of examinee *i*, that is, 1 indicates examinees’ mastery of attribute *k*, and otherwise 0.

The DINA model is a completely non-compensatory model, which requires that the examinees must master all the attributes required by the item for correctly answering. As long as any one of them is not mastered, it will lead to a wrong answer or a very low probability of correct answer. For the value of the ideal response $\eta_{\text{ij}}=\prod_{k=1}^{K}\alpha_{i\,k}^{q^{j\,k}}$, a value of 1 indicates that the examinee *i* has mastered all the attributes measured by the item *j*, while a value of 0 means that the examinee has not fully mastered the attributes measured by the item *j*. The corresponding probabilities of correct answer to this item are (1−*s*_*j*_) and *g*_*j*_ respectively. The formula of DINA model is as follows (Junker and Sijtsma, 2001)

(1) $$\text{P}\left(X_{i\,j}=1|\alpha_{i}\right)={\left(1-s_{j}\right)}^{\eta_{\text{ij}}}g_{j}^{\left(1-\eta_{\text{ij}}\right)}.$$

The DINO model is the compensatory model. As long as the examinees have mastered any of the attributes measured by the item, they can have a higher probability of correctly answering. For the value of the ideal response $\varpi_{i\,j}=1-\prod_{k=1}^{K}{\left(1-\alpha_{i\,k}\right)}^{q_{j\,k}}$ is 1, it means that the examinees have mastered at least one attribute measured by item *j*. A value of 0 indicates that the examinees have not mastered all the attributes of item *j*. The formula of the DINO model is as follows (Templin and Henson, 2006)

(2) $$\text{P}\left(X_{i\,j}=1|\alpha_{i}\right)={\left(1-s_{j}\right)}^{\varpi_{i\,j}}g_{j}^{\left(1-\varpi_{i\,j}\right)}.$$

where *s*_*j*_ is the slip probability for the examinees of the ideal response with value 1 on item *j*, and *g*_*j*_ is the guessing probability for the examinees with value 0 on item j.

Like the DINA model, R-RUM is a non-compensatory model, which is a simplified unified model of reparameterization. The baseline parameter $\pi_{j}^{*}$ indicates the positive response probability for examinees who have mastered all the attributes required by item *j*. The values are all between 0 and 1. The penalty parameter $r_{j\,k}^{*}$ for not possessing the *k*th attribute is defined at the level of interaction between the item and the attributes and reflects the importance of attribute *k* on item *j*. The formula of R-RUM is as follows (Hartz, 2002)

(3) $$P\left(X_{i\,j}=1|\alpha_{i}\right)=\pi_{j}^{*}\prod_{k=1}^{K}r_{j\,k}^{*\left(1-\alpha_{i\,k}\right)\,q_{j\,k}}.$$

For simplify, the correct answer probability P(*X*_*ij*_ = 1|α_*i*_) is denoted by *P*_*j*_(α_*i*_), where α_*i*_ is the knowledge state of the examinee *i*.

### Kullback-Leibler Information Distance Between Classes

Considering the existing cognitive diagnosis item bank, attribute vectors of all items in the item bank have been specified (Wang et al., 2020), and the parameters of each item have been estimated by the parameter estimation algorithm of cognitive diagnosis model. The correct answer probability *P*_*j*_(α_*c*_) of knowledge state α_*c*_ on item *j* can be calculated by item attribute vector *q*_*j*_ and item parameters, where α_*c*_ is a knowledge state of the examinees and is an element of the universal set of latent classes. Let M be the size of the item bank. Kullback-Leibler (K-L; Cover and Thomas, 2006; Debeer et al., 2020) information quantity or K-L distance is the most commonly used to measure the distance between any two probability distributions *P*_*j*_(α_*u*_) and *P*_*j*_(α_*v*_) for two knowledge states α_*u*_ and α_*v*_. Formally, item *j* is defined as the K-L distance of the item response probability distributions under the knowledge states of α_*u*_ and α_*v*_

(4) $$D_{K-L}\,\left(\alpha_{u},\alpha_{v},\text{j}\right)=P_{j}\,\left(\alpha_{u}\right)\,\log\left[\frac{P_{j}\,\left(\alpha_{u}\right)}{P_{j}\,\left(\alpha_{v}\right)}\right]+\left(1-P_{j}\,\left(\alpha_{u}\right)\right)\,\log\left[\frac{\left(1-P_{j}\,\left(\alpha_{u}\right)\right)}{\left(1-P_{j}\,\left(\alpha_{v}\right)\right)}\right].$$

In fact, *D*_*K–L*_ is the expectation of the function of the logarithmic likelihood ratio of probability distributions *P*_*j*_(α_*u*_) and *P*_*j*_(α_*v*_). Although this amount of information is called the distance between the two distributions, and it does have statistical significance for distance measurement, that is, with the increase of *D*_*K–L*_, it is easier to distinguish the two distributions statistically (Rao, 1962). But it is not symmetrical, that is, *D*_*K*−*L*_(α_*u*_,α_*v*_)≠*D*_*K*−*L*_(α_*v*_,α_*u*_).

Kullback-Leibler distance is often used for computer adaptive testing or cognitive diagnostic computer adaptive testing. For instance, Chang and Ying (1996) firstly suggested that K-L distance instead of Fisher information should be used as a more effective item selection index in computer adaptive testing based on one-dimensional IRT model. Madigan and Almond (1995) use K-L distance for test selection strategy of belief networks. Tatsouka and Ferguson (2003) use K-L distance and Shannon entropy for sequential item selection and use it in cognitive diagnostic computer adaptive testing. Different from the amount of Fisher information, the K-L distance does not require that the parameter space must be continuous, so it is suitable for CDM where the attribute pattern is discrete.

### Test Assembly Using Mixed-Integer Linear Programming

In cognitive diagnosis, the probability of correct answer or the expected vector of item response of knowledge state α_*c*_ on test length of *J* in a test isP(α_*c*_) = (*P*_1_(α_*c*_),*P*_2_(α_*c*_),…,*P*_*J*_(α_*c*_)). For knowledge state α_*c*_, the P(α_*c*_) can be regarded as the center of the class. In pattern recognition or clustering methods, the method of maximum distance between classes can usually be used for classification. If the cognitive diagnostic test can maximize the distance between the class centers of all potential classes α_*c*_ ∈ *Q*_*s*_, where *Q*_*s*_ is the universal set of latent classes, it is easier to classify knowledge states. It is just like in a jigsaw puzzle, if there is a big difference between the sub-images, the difficulty of completing the puzzle will be correspondingly lower.

In order to characterize the distinguishing power of item *j* to knowledge states α_*u*_ and α_*v*_, the following is *D*_*K–L*_ as its metric index. For any α_*u*_ and α_*v*_, the discrimination power matrix or K-L distance matrix *D*_*j*_ = (*D*(α_*u*_,α_*v*_,*j*)) is obtained. If the cardinality (i.e., the number of elements) of *Q*_*s*_ is *T*, we know that the number of rows or columns is *T* in *D*_*j*_. In order to use the mixed-integer linear programming for test construction, it is necessary to vectorize the matrix *D*_*j*_ into a single stacked column vector. That is, the sequence of rows in this matrix is composed of a long vector, and then transpose the row vector to get the stacked column vector, which is denoted as *V*_*j*_ = *Vec*(*D*_*j*_). When the matrix *D*_*j*_ is vectorized, we remove the main diagonal elements because these values are zeros. For each item in the item bank, *V*_*j*_ can be calculated, and the matrix *V* = (*V*_1_,*V*_2_,…,*V*_*M*_) composed of all the items can be obtained, where *M* is the number of items in the item bank. Based on the mixed-integer linear programming model, we will give a linear programming model which takes into account the mean value of the distance between all classes and maximizes the minimum distance between classes:

(5) $$\text{min}\,\left(f_{1}\,x+f_{2}\,y\right),$$

Subject to

V⁢x+y≥b,

$$1^{T}\,x=J,$$

*x*_*j*_ ∈ {0,1}, j = 1, 2, …, M,

y∈R.

Among them, *f*_1_ = (*f*_11_,*f*_12_,…,*f*_1*M*_)^*T*^, where $f_{1\,j}=-\sum_{v=1}^{T\,\text{T}-1}V_{v\,j}/\left(T\,\left(\text{T}-1\right)\right)$. The negative of *f*_*1j*_ is used to convert a maximization problem into a minimization one. Here, $f_{2}=J\,\sum_{u=1}^{T\,\left(T-1\right)}\sum_{v=1}^{M}V_{u\,v}/\left(\text{TM}\,\left(\text{T}-1\right)\right)$ is the weight of y, *x* = (*x*_1_*x*_2_…*x*_*M*_)^*T*^, where *x*_1_*x*_2_⋯*x*_*M*_ is the 0-1 vector in the decision vector of linear programming, and the value of the *x*_*j*_ indicated whether the test contains the item *j*. If *x*_*j*_ = 1, it means that the test contains the item *j*, otherwise it does not include the item *j*, b = (*b*_1_,*b*_2_,…,*b*_*T*(*T*−1)_)^*T*^ represents the lower limit of K-L distance for all pairs of knowledge states. You can set the bounded distance $b_{t}=J\,\sum_{j=1}^{M}\left(V_{t\,j}\right)/M$, which is the average value of the distance between classes of *J* items in the item bank. 1^*T*^*x* = *J* represents the test length constraint, where 1^*T*^is a *M*-dimensional column vector with all elements 1, and *J* is the test length. *y* captures the difference between the *t-*th pair inter-class distance *V*_(*t*)_*x* and the target distance *b*_*t*_, where *V*_(*t*)_is row t in *V*. Then, adding y to the constraint condition, and adding *f*_2_*y* to the objective function, is to maximize the minimum inter-class distance y. For example, if the components in ***b*** are equal, and *V*_(*t*)_*x* is the smallest of all the distances between classes, if *V*_(*t*)_*x* < *b*_*t*_, then *V*_(*t*)_*x* can at least add *b*_*t*_−*V*_(*t*)_*x* to satisfy the constraint. Because the average distance between other classes is larger than *V*_(*t*)_*x*, *V*_(*t*)_*x* needs to add *b*_*t*_−*V*_(*t*)_*x* to reach the constraint. And minimizing *f*_2_*y* in the objective function is minimizing *f*_2_(*b*_*t*_−*V*_(*t*)_*x*).Because f_2*y*_ is positive and *b*_*t*_ fixed, that is maximizing *V*_(*t*)_*x* which is the minimum inter-class distance between classes. In the objective function, we also consider the *f*_1_*x*, linear programming model at the same time, that is, to maximize the distance between all classes, because the model also contains 0-1 vector *x* and real vector y, so this linear programming model is a typical mixed-integer or mixed 0-1 linear programming model, which can be solved by intlinprog function in Matlab2015a. For the source codes, we provided a user-friendly code in MATLAB into a public repository at the website: https://github.com/JXNU-EduM/MMD-Test-Assembly-for-CD/.

### Simplify of K-L Distance Matrix

The distance index*D*_*K*−*L*_ in this study needs to be calculated for mixed-integer linear programming, so it is necessary to process the distance index matrix with vectorizing, transposing and merging. In the case of no hierarchical structure of attributes, there are *T* = 2^*K*^ possible mastery modes for *K* attributes, and there are *M* items in the item bank. The size of the distance matrix of *M* items on the 2^*K*^ attribute mastery patterns after vectorizing, transposing and merging is M*2^*K*^(2^*K*^−1). If *M* is 300 and *K* is 4, the size of the distance matrix is 300^∗^240. Although the size of the matrix is within the acceptable range, the amount of calculation for mixed-integer linear programming is a little large, so if possible, the distance matrix should be simplified.

**FIGURE 1:**
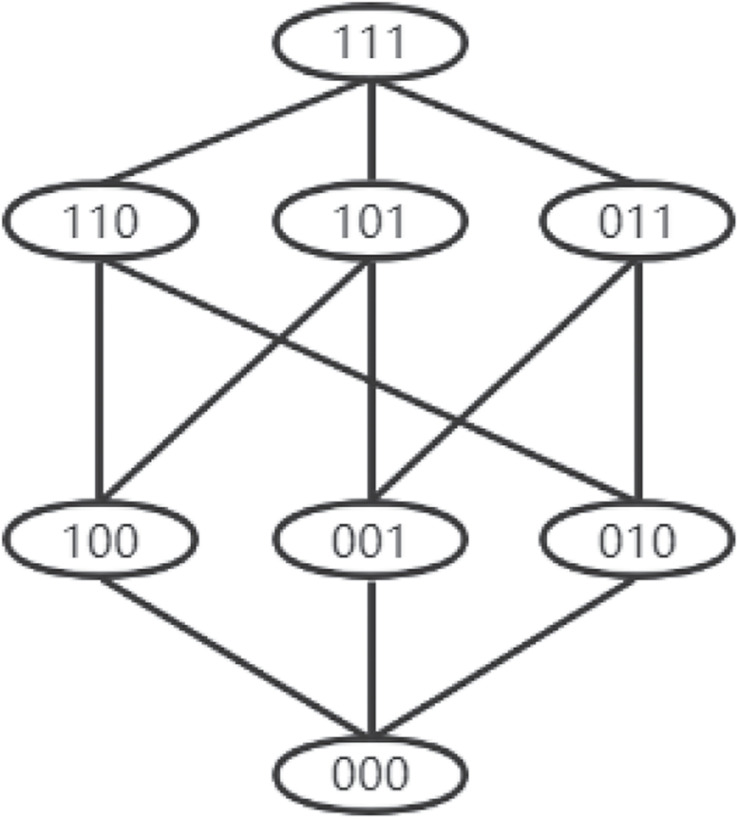
Partial relation for eight possible attributes mastery patterns.

If the u-th row and v-column element in *D*_*j*_ is denoted by *D*_*juv*_, and the corresponding element in *V*_*j*_ is denoted by *V*_*juv*_. *D*_*juv*_ or *V*_*juv*_ is the discriminating power for these two different knowledge states of α_*u*_ and α_*v*_, and one condition for the smallest difference between the two knowledge states is that there is a k-th attribute in the two attribute mastery patterns, which makes the k-th attribute mastery status of the two patterns different, and all mastery status except k are exactly the same. If only the discriminating power among attribute patterns with the least difference for the item is considered when vectorizing the distance matrix, the *V*_*juv*_ can be simplified. In the following, the distance matrix index corresponding to the simplified *V*_*juv*_ is recorded as SD_*K*−*L*_. According to the characteristics of attribute patterns, we know that if the number of attributes is *K* and a certain attribute pattern is given, there are *K* attribute patterns with the least difference from it. Because of the asymmetry of the distance between α_*u*_ and α_*v*_, that is, the D_*K*−*L*_ distance from α_*u*_ and α_*v*_ is different from that from α_*v*_ to α_*u*_, both *D*_*juv*_ and *D*_*jvu*_ should be considered. If the number of attributes is three and the attributes are independent and without hierarchical structure, there are eight possible attribute mastery patterns, as shown in Figure 1: the difference of attribute patterns with connections between adjacent levels is the smallest. Thus, only 24 elements needed to be considered in *D*_*j*_ is obviously smaller than the number of non-diagonal elements in *D*_*j*_, which can greatly save the computational cost.

## Study Design

Some main factors that may affect the efficiency of constructing test assembly should be considered: cognitive diagnosis model (the DINA model, the DINO model, and the R-RUM), attribute correlation coefficient (0 and 0.5), the number of examinees was fixed at 10000, the size of item bank was fixed at 300, the number of measured attributes was fixed at 4. Attribute correlation coefficient is zero, implying that the attributes were independent of each other, and the knowledge state was distributed evenly. Under each condition, the experiment was repeated for 200 times.

Assuming that the test measured *K* attributes, there are at most 2^*K*^−1 possible item attribute vectors. First of all, all possible item attribute binary vectors were converted to decimal as 1, 2,…, 2^*K*^−1, and then 300 random integers in the range [1,  2^*K*^−1] were randomly generated. Item attribute vectors of 300 items with corresponding numbers were selected to form the Q-matrix for an item bank. Item parameters of each item were randomly generated from specified distributions. The DINA and DINO models have the guessing and slip item parameters, which are randomly generated from a uniform distribution U (0.05, 0.4). Meanwhile the R-RUM also has the baseline and penalty parameters, which are respectively randomly generated from the uniform distribution U (0.75, 0.95) and U (0.2, 0.95). These were the same as the experimental design of Henson and Douglas (2005).

When the examinees are simulated, two aspects need to be considered: one is attribute mastery status α_ki_ at the k-th attribute for the i-th examinee and the other is the correlation coefficient between attributes, denoted by ρ. Multivariate normal distribution can be used to simulate latent ability, ${\tilde{\alpha }}_{i}∼\text{MVN}\,\left(0,\Sigma_{K*K}\right)$, where **0** is the zero vector with the length of *K* and **Σ** is the correlation matrix

(6) $$\Sigma =\left[\begin{array}{ccc} 1 & \cdots & \rho \\ \vdots & \ddots & \vdots \\ \rho & \cdots & 1 \end{array}\right].$$

In this study, the value of ρ is 0 (independent structure) or 0.5. After getting the value of ${\tilde{\alpha }}_{\text{ki}}$, we need to discretize it. The strategy of discretization of α_*ki*_ is

(7) $$\alpha_{\text{ki}}=\left\{ \begin{array}{c} 1\;i\,f\,{\tilde{\alpha }}_{k\,\text{i}}\geq 0,\\ 0\;o\,t\,h\,e\,r\,w\,i\,s\,e. \end{array}\right.$$

Two groups of 10000 examinees were simulated. One group of examinees was used to calculate the empirical distribution of knowledge state, which will be applied as the prior distribution for compute the posterior mode in the classification of the other group. We have not changed this condition for the repetition of the study of Henson and Douglas (2005). If a lager sample is available for the calibration of item bank, the empirical distribution of attribute patterns from the large sample will be applied as the prior distribution to computing the posterior mode in the classification of examinees who have taken the tests constructed from the calibrated item bank.

For a set of given attribute mastery pattern, PX_ij_ = 1|α_i_ depending on the selected model is the probability of correct response to item *j* for examinee *i* with attribute mastery pattern α_*i*_. We supposed *u* was randomly generated from a uniform distribution U (0, 1). The item response of the *i*th examinee on item *j* can be obtained by discretizing the probability matrix

(8) $$X_{i\,j}=\left\{ \begin{array}{cc} 1 & ifu\leq P\left(X_{i\,j}=1|\alpha_{i}\right)\\ 0 & o\,t\,h\,e\,r\,w\,i\,s\,e. \end{array}\right.$$

Since the item parameters were known, the examinees’ item responses on the selected items could be simulated, and then the examinees were classified by maximum posterior estimation, and then attribute correct rate (ACR) and pattern correct rate (PCR) could be calculated. The formulas of ACR and PCR are as follows

(9) $$\text{ACR}=\frac{1}{N\,\text{K}}\sum_{k=1}^{K}\sum_{i=1}^{N}I\left(\alpha_{\text{ik}}={\hat{\alpha }}_{\text{ik}}\right),$$

and

(10) $$\text{PCR}=\frac{1}{N}\sum_{\text{i}=1}^{N}I\left(\alpha_{i}={\hat{\alpha }}_{i}\right).$$

In the above two expressions, *N* and *K* represent the number of examinees and the number of attributes, respectively, and *I*(*x* = y) is an indicative function, which is defined as follows: when *x* = y, *I*(*x* = y) = 1, otherwise it is 0. The attribute correct rate (ACR) is the proportion of examinees whose estimated attribute status is equal to the simulated or true attribute status, while the pattern correct rate (PCR) is the proportion of examinees whose estimated attribute patterns is equal to the simulated or true attribute patterns. These two indices are commonly used in the simulation study for evaluating the correct classification rates for attributes or attribute patterns. The higher PCR and ACR for a test construct method implies that it yields considerably higher correct classification rates.

The *D*_*K–L*_ distance was used as the inter-class distance, and the mixed-integer linear programming is used to maximize the minimum inter-class distance with additional constraints. The test length is 20 for all test design. The first constraint was no constraint (No Constraints, NC), which directly used the greedy algorithm to construct test, and did not set any constraints based on the CDI or MDD. The second constraint was item-level constraint (Item Constraints, IC), which controls the number of items that measure a specific number of attributes for test assembly. According to the suggestion of Henson and Douglas (2005), among the 20 items that measure a total of 4 attributes, 9 items measured three attributes, 7 items measured two attributes, and the remaining 4 items measured one attribute. The third constraint was the attribute number constraint (Attribute Constraints, AC), which required that each attribute must be measured at least 7 times in a test with four attributes and 20 items.

## Study 1: Comparison Between the Proposed Method and Its Simplification

The proposed method uses mixed-integer linear programming to maximize the minimum inter-class distance between classes and comprehensively to consider the overall amount of information in order to achieve better test assembly quality. However, when the number of attributes measured was four, the calculation of the distance matrix D_*K*−*L*_ after vectorizing by the new method was a bit large, so when using the new method to construct test assembly, the distance matrix needs to be simplified. The test assembly method using the original and simplified matrices were denoted by D_*K*−*L*_ and SD_*K*−*L*_, respectively. In fact, the simplification of the distance matrix will reduce the constraints of mixed-integer linear programming. The simplified matrix aims to discriminate similar attribute patterns, but whether it will lose the amount of information, if it is true, the size of the loss still needs to be verified.

### Research Purpose

The purpose of this study is to verify whether the simplified distance matrix will lose information and lead to poor results. Since this study only considered the effect of simplified constraints on the efficiency of the MMD test assembly method, a single factor or one-way analysis of variance (ANOVA) can be performed on the two groups of ACR and PCR before and after the simplification in order to measure the impact of simplified constraints on ACR and PCR. In addition, the mean of ACR or PCR before and after simplification and the index of constructing test assembly time (in seconds) need to be taken into account.

### Experimental Steps

In order to achieve the purpose of this study, the experiment was designed according to the following steps:

(1) According to the design of Section 3 (four attributes were considered), we simulate two groups of examinees, in which one group was used to calculate the prior distribution, and the other group was used for classification. We simulate the Q matrix and item parameters in the item bank, and simulate the observed complete item response matrix of all examinees on all items in the item bank.
(2) Calculate the D_*K*−*L*_ distance and the simplified D_*K*−*L*_ distance of all items on all possible attribute mastery patterns.
(3) Choose the items according to the strategies of no restriction, attribute restriction and item restriction;
(4) Take out the response matrix of all the items on the corresponding test according to the test items generated by the test assembly algorithm;
(5) Estimate the knowledge state of the examinees and calculate the PCR and ACR, according to the selected response matrix, and repeat experiments for a total of 200 times.
(6) A one-way analysis of variance was performed on the data before and after the simplification. The specific steps of the analysis method were as follows:

We conduct a statistical test to compare the means for the PCR and ACR from two methods with the null hypothesis *H*_*0*_: The simplified constraint has no significant effect on the ACR and PCR of the MMD test assembly method.

In order to express the differences of the means for the PCR or ACR from two methods, the simplified ACR (the same for PCR analysis) is combined into a two-column matrix *Y*_*ij*_, i = 1,2; j = 1,2, …, n. The sum of samples is set to $Y_{i.}=\sum_{j=1}^{n}Y_{i\,j}$, and the sample mean is ${\bar{Y}}_{i}=\frac{1}{n}\,\sum_{j=1}^{n}Y_{i\,j}$, then the calculation formula for the total mean of the samples is

(11) $$\bar{Y}=\frac{1}{n}\,\sum_{i=1}^{2}\sum_{j=1}^{n}Y_{i\,j}.$$

The sum of squares of deviations is an indicator of the degree of dispersion of all data. If the assumption *H*_*0*_ holds, the simplified constraint will have no significant effect on ACR or PCR, and then the difference of data in *Y*_*ij*_ is caused by other random factors. If the assumption is not true, in addition to random factors, the data difference in *Y*_*ij*_ also has the influence of simplified constraints. If the influence of simplified constraints is much greater than that of random factors, the simplified constraints should be considered to have a significant impact on ACR or PCR, otherwise it is considered to have no significant impact. Among them, the calculation formulas for the sum of squares between groups *S*_*A*_ and the random error sum of squares (or sum of squares within groups) *S*_*E*_ are

(12) SA=∑i=12n(Y¯i-Y¯),2

and

(13) SE=∑i=12∑j=1n(Yi⁢j-Y¯i).2

In this study, only one factor was considered, so the degree of freedom of *S*_*A*_ was 1, and the total observation data was set to 2n, then the degree of freedom of *S*_*E*_ was 2n-2. From this, the formula for the one-way analysis of variance F-test can be calculated

(14) $$F=\frac{S_{A}}{S_{E}/\left(2\,n-2\right)}.$$

After the observed value of F was obtained by analyzing and calculating from the data, we can usually choose a significant level of 0.05 or 0.01 according to the accuracy rate requirements. Then, the p-value was computed based on the observed value of F. Finally, the p-value is compared with 0.05 or 0.01 to decide whether to accept the null hypothesis. In this study, the significance level was set to 0.05.

### Experimental Results

Tables 1–3 are results of the one-way analysis of variance of ACR and PCR obtained by the simplified and non-simplified constraint MMD test assembly method under the DINA model, the DINO model and the R-RUM, respectively. It can be seen that the p-value of DINA and DINO models are greater than 0.05 in all relevant cases, indicating that there is no significant difference in ACR or PCR between before and after the simplified constraints. However, the p-value of item constraints on the R-RUM is lower than 0.05, indicating that there is a significant difference in ACR or PCR between before and after the simplified constraints. It shows whether the constraints are simplified or not has little effect on the efficiency of the MMD constructing test assembly, except under the item constraints on the R-RUM.

**TABLE 1:** Single factor analysis of variance for simplified and non-simplified constraints under the DINA model.

Correlation	Accuracy	Constraints	*S*_*A*_	*S*_*E*_	F	*p*-value
0	ACR	NC	5.3222E-06	0.0054	0.3942	0.5304
		IC	3.4223E-09	0.0168	0.0001	0.9928
		AC	6.7185E-06	0.0059	0.4556	0.5001
	PCR	NC	6.9139E-05	0.0604	0.4559	0.4999
		IC	9.8010E-07	0.0966	0.0040	0.9494
		AC	6.4883E-05	0.0654	0.3951	0.5300
0.5	ACR	NC	9.8533E-06	0.0052	0.7510	0.3867
		IC	4.4944E-08	0.0177	0.0010	0.9747
		AC	2.9618E-06	0.0050	0.2346	0.6284
	PCR	NC	1.2589E-04	0.0619	0.8099	0.3687
		IC	4.6923E-07	0.1320	0.0014	0.9700
		AC	3.4047E-05	0.0592	0.2290	0.6326

**TABLE 2:** Single factor analysis of variance for simplified and non-simplified constraints under the DINO model.

Correlation	Accuracy	Constraints	*S*_*A*_	*S*_*E*_	F	*p*-value
0	ACR	NC	1.1516E-05	0.0060	0.7602	0.3838
		IC	1.2100E-10	0.0163	0.0000	0.9986
		AC	6.0639E-06	0.0060	0.4025	0.5262
	PCR	NC	1.0040E-04	0.0670	0.5961	0.4405
		IC	5.0625E-08	0.0939	0.0002	0.9883
		AC	6.6831E-05	0.0668	0.3985	0.5282
0.5	ACR	NC	5.8443E-06	0.0054	0.4343	0.5103
		IC	3.8813E-07	0.0154	0.0100	0.9202
		AC	2.2801E-06	0.0054	0.1688	0.6814
	PCR	NC	7.1234E-05	0.0658	0.4309	0.5119
		IC	2.6732E-06	0.1209	0.0088	0.9253
		AC	1.8966E-05	0.0654	0.1154	0.7343

**TABLE 3:** Single factor analysis of variance for simplified and non-simplified constraints under the R-RUM model.

Correlation	Accuracy	Constraints	*S*_*A*_	*S*_*E*_	F	*p*-value
0	ACR	NC	3.0360E-07	0.0082	0.0147	0.9035
		IC	4.1598E-04	0.0114	14.4606	0.0002
		AC	8.2369E-08	0.0085	0.0039	0.9505
	PCR	NC	4.1209E-06	0.0854	0.0192	0.8898
		IC	1.3195E-03	0.0886	5.9273	0.0153
		AC	1.6512E-06	0.0882	0.0075	0.9313
0.5	ACR	NC	1.7222E-09	0.0051	0.0001	0.9908
		IC	1.9847E-04	0.0058	13.6922	0.0002
		AC	2.4602E-07	0.0051	0.0193	0.8896
	PCR	NC	2.3040E-07	0.0584	0.0016	0.9684
		IC	1.1219E-03	0.0507	8.8060	0.0032
		AC	3.2580E-06	0.0586	0.0221	0.8818

Tables 4–6 respectively give a detailed comparison of simplified and non-simplified constraints in terms of ACR and PCR under each condition of the DINA model, the DINO model and the R-RUM. The sixth column of the tables indicates that the accuracy rate of simplified constraints higher than the accuracy rate of non-simplified constraints.

**TABLE 4:** Comparison of simplified and non-simplified constraints under the DINA model.

Correlation	Accuracy	Constraints	*D*_*K–L*_	*SD*_*K–L*_	*SD*_*K*−*L*_ Outperforms *D*_*K–L*_	Time for *D*_*K–L*_ (seconds)	Time for *SD*_*K–L*_ (seconds)
0	ACR	NC	0.9798	0.9795	0.4500	1.9195	0.7373
		IC	0.9463	0.9463	0.9700	0.3661	0.1022
		AC	0.9796	0.9793	0.4250	1.9718	0.7971
	PCR	NC	0.9260	0.9251	0.4500	1.9195	0.7373
		IC	0.8356	0.8357	0.9800	0.3661	0.1022
		AC	0.9253	0.9245	0.4150	1.9718	0.7971
0.5	ACR	NC	0.9810	0.9807	0.4350	1.8395	0.7196
		IC	0.9489	0.9489	0.9800	0.3429	0.0909
		AC	0.9808	0.9806	0.4850	1.9187	0.7774
	PCR	NC	0.9292	0.9281	0.4400	1.8395	0.7196
		IC	0.8349	0.8350	0.9800	0.3429	0.0909
		AC	0.9286	0.9280	0.4800	1.9187	0.7774

**TABLE 5:** Comparison of simplified and non-simplified constraints under the DINO model.

Correlation	Accuracy	Constraints	*D*_*K–L*_	*SD*_*K–L*_	*SD*_*K*−*L*_ Outperforms *D*_*K–L*_	Time for *D*_*K–L*_ (seconds)	Time for *SD*_*K–L*_ (seconds)
0	ACR	NC	0.9794	0.9790	0.4300	1.7908	0.8185
		IC	0.9457	0.9457	0.9800	0.3470	0.1052
		AC	0.9793	0.9791	0.4700	1.8486	0.8369
	PCR	NC	0.9246	0.9236	0.4750	1.7908	0.8185
		IC	0.8346	0.8346	0.9800	0.3470	0.1052
		AC	0.9245	0.9237	0.4800	1.8486	0.8369
0.5	ACR	NC	0.9814	0.9811	0.4450	1.7821	0.8107
		IC	0.9494	0.9495	0.9950	0.3277	0.0955
		AC	0.9811	0.9810	0.5150	1.8327	0.8403
	PCR	NC	0.9305	0.9297	0.4450	1.7821	0.8107
		IC	0.8358	0.8360	0.9950	0.3277	0.0955
		AC	0.9297	0.9292	0.5300	1.8327	0.8403

**TABLE 6:** Comparison of simplified and non-simplified constraints under the R-RUM model.

Correlation	Accuracy	Constraints	*D*_*K–L*_	*SD*_*K–L*_	*SD*_*K*−*L*_ Outperforms *D*_*K–L*_	Time for *D*_*K–L*_ (seconds)	Time for *SD*_*K–L*_ (seconds)
0	ACR	NC	0.9514	0.9514	0.5250	3.3500	0.8915
		IC	0.9332	0.9311	0.2600	0.3594	0.1072
		AC	0.9513	0.9513	0.5150	3.4402	0.9404
	PCR	NC	0.8270	0.8272	0.5500	3.3500	0.8915
		IC	0.7816	0.7780	0.3750	0.3594	0.1072
		AC	0.8267	0.8269	0.5500	3.4403	0.9404
0.5	ACR	NC	0.9590	0.9590	0.4950	3.3598	0.8921
		IC	0.9457	0.9443	0.2750	0.3369	0.1024
		AC	0.9590	0.9589	0.5000	3.4420	0.9420
	PCR	NC	0.8495	0.8495	0.5000	3.3598	0.8921
		IC	0.8115	0.8081	0.3500	0.3369	0.1024
		AC	0.8494	0.8492	0.5050	3.4420	0.9420

It can be seen from Table 4 that under the DINA model, when the MMD test assembly simplifies the constraints, the overall efficiency is less than 50% although the efficiency of the simplified constraints is higher than that of the non-simplified constraints. Therefore, the simplification of the distance matrix will indeed lose information. From the perspective of the overall mean, the loss of information has a relatively low impact on the efficiency of the test assembly. This conclusion is similar to the results of the one-way analysis of variance. In terms of average time consumption, simplifying the constraints will increase the operating efficiency by 2 to 4 times. Comparing with the information of lost by the simplified constraints, the improvement of the operating efficiency is considerable. Therefore, the simplified constraints on the distance matrix are feasible.

Tables 5, 6 shows that the efficiency of the simplified constraints is higher than that of the non-simplified constraints, the efficiency is more than 50% or close to 50% under the attribute constraints, but the overall situation is still lower than the non-simplified constraints and the difference is still small under the DINO model and R-RUM. In terms of time-consuming, the time-consuming for these two models is similar to that under the DINA model, but simplifying the constraints will still increase the operating efficiency by 2 to 4 times on average, so a similar conclusion can be obtained with the DINA model.

## Study 2: Comparison Between Simplified MMD Method and CDI Method

### Experimental Purpose

Study 1 has verified that the simplified constraints on the distance matrix is feasible, so how the new method itself compares with the famous method needs to be discussed further. In order to compare the simplified MMD test assembly method and the CDI method (Henson and Douglas, 2005), we performed the second simulation experiments by using the similar condition settings as the study of Henson and Douglas (2005). It should be noted that eight attributes were considered in the second simulation study for exploring the performance of the simplified MMD test assembly method under different conditions.

### Experimental Steps

Conduct the simulation experiment as follows:

(1) According to the design of the first study, we simulated two groups of examinees, one of groups was used to calculate the prior distribution and the other was used for classification. The Q matrix and item parameters in the item bank and observed complete item response matrix of all possible attribute mastering patterns on all items in the item bank were simulated;
(2) Calculate the CDI and SD_*K*−*L*_ of all items;
(3) Construct cognitive diagnostic test using the random way, the CDI method, or the simplified MMD method, according to the three strategies of no constraints, attribute constraints and item constraints;
(4) Take out the response matrix of all the items on the corresponding test according to the test items generated by the test assembly algorithms;
(5) Estimate the knowledge state of the examinees and calculate the PCR and ACR, according to the selected response matrix, and repeat experiments for a total of 200 times.

### Experimental Results

Table 7 shows the average accuracy rate of each condition under measuring four attributes with the DINA model. In the table, CDI represents the CDI test assembly method, SD_*K*−*L*_ is the simplified MMD test assembly method, and Random represents random test assembly. Analyzing the data in Table 7 shows that the new method has a higher improvement compared with the CDI method. In terms of the three constraints, the overall accuracy rate of the attribute constraints is slightly higher than the other two constraints, and the accuracy rate for the item constraints is the worst. Under the condition of item constraints, the ACR and PCR of CDI, MMD, and random test assembly method are lower than the other two constraints.

**TABLE 7:** The accuracy rate of each condition for four attributes under the DINA model.

Correlation	Accuracy	Constraints	Random	CDI	SD_*K*−*L*_
0	ACR	NC	0.8443	0.9692	0.9795
		IC	0.8257	0.9173	0.9463
		AC	0.8439	0.9730	0.9793
	PCR	NC	0.5803	0.8886	0.9251
		IC	0.5507	0.7498	0.8357
		AC	0.5806	0.9054	0.9245
0.5	ACR	NC	0.8801	0.9733	0.9807
		IC	0.8680	0.9401	0.9489
		AC	0.8798	0.9756	0.9806
	PCR	NC	0.6588	0.9013	0.9281
		IC	0.6377	0.8022	0.8350
		AC	0.6587	0.9106	0.9280

Table 8 shows the comparison of the accuracy rate of each method when the number of attributes under the DINA model is four in the 200 repeats. Among them, the last column represents the proportion of MMD test assembly method with SD_*K*−*L*_ distance as the class distance index more efficient than CDI in the 200 simulation repeats. The fourth or fifth column respectively represents the proportion of CDI test assembly method or MMD test assembly method with SD_*K*−*L*_ distance more efficient than the random test assembly method across 200 repetitions.

**TABLE 8:** Comparison of the accuracy rate of each method for four attributes under DINA model.

Correlation	Accuracy	Constraints	CDI Outperforms Random	SD_*K*−*L*_ Outperforms Random	SD_*K*−*L*_ Outperforms CDI
0	ACR	NC	1.0000	1.0000	0.9150
		IC	0.9950	1.0000	0.9500
		AC	1.0000	1.0000	0.8850
	PCR	NC	1.0000	1.0000	0.9250
		IC	1.0000	1.0000	0.9550
		AC	1.0000	1.0000	0.8600
0.5	ACR	NC	1.0000	1.0000	0.8800
		IC	1.0000	1.0000	0.9050
		AC	1.0000	1.0000	0.8500
	PCR	NC	1.0000	1.0000	0.8850
		IC	1.0000	1.0000	0.9300
		AC	1.0000	1.0000	0.8500

It can be seen from Table 8 that the MMD test assembly method with SD_*K*−*L*_ distance as an index is stable under various conditions. In the existing conclusions, as the correlation increasing, the accuracy rate of the MMD test assembly method the CDI method based on SD_*K*−*L*_ distance increases. Therefore, as the correlation increasing, the gap between the two methods will shrink. Thus, comparing with random test assembly, the average value of each method is greater than the random method. However, in the 200 simulation repeats, the CDI test assembly method is occasionally outperformed by the random test assembly method, which is similar to the simulation results of Henson and Douglas (2005).

On the whole, the result of CDI test assembly method is slightly different from that of Henson and Douglas (2005) in comparison with random test assembly method under measuring four attributes, because the random test assembly method itself is uncertain. In addition, the case that the accuracy rate of CDI test assembly method is lower than that of the random method is concentrated under the item constraints.

Table 9 shows the comparison of several test assembly methods for 200 repetitions under DINO model. From the data in Table 9, it can be seen that the MMD method is still superior to CDI method, and the MMD method with SD_*K*−*L*_ distance as the distance index does not have the situation that the average accuracy rate is lower than CDI method.

**TABLE 9:** Comparison of the accuracy rate of each method for four attributes under DINO model.

Correlation	Accuracy	Constraints	Random	CDI	SD_*K*−*L*_
0	ACR	NC	0.8428	0.9687	0.9790
		IC	0.8256	0.9173	0.9457
		AC	0.8429	0.9728	0.9791
	PCR	NC	0.5779	0.8867	0.9236
		IC	0.5510	0.7490	0.8346
		AC	0.5786	0.9040	0.9237
0.5	ACR	NC	0.8791	0.9739	0.9811
		IC	0.8696	0.9409	0.9495
		AC	0.8792	0.9758	0.9810
	PCR	NC	0.6566	0.9033	0.9297
		IC	0.6406	0.8041	0.8360
		AC	0.6573	0.9114	0.9292

Table 10 shows the comparison of the accuracy rate of each method when the number of attributes is four across replications under the DINO model. It can be seen from Table 10, the MMD method with SD_*K*−*L*_ distance as the distance index has slightly better accuracy rate than the CDI method under the condition of unconstrained and attribute constraints, respectively. However, the accuracy rate of the MMD test assembly method under the item constraints is better than under the other two constraints. In the existing conclusions, with the increase of correlation, the accuracy rate of MMD test assembly method with SD_*K*−*L*_ distance as the index decreased while that of the CDI method increased. Therefore, with the increase of correlation, the gap between the two methods narrowed.

**TABLE 10:** Comparison of the accuracy rate of each method for four attributes under the DINO model.

Correlation	Accuracy	Constraints	CDI Outperforms Random	SD_*K*−*L*_ Outperforms Random	SD_*K*−*L*_ Outperforms CDI
0	ACR	NC	1.0000	1.0000	0.9250
		IC	0.9850	1.0000	0.9800
		AC	1.0000	1.0000	0.8750
	PCR	NC	0.9950	1.0000	0.9350
		IC	0.9900	1.0000	0.9800
		AC	1.0000	1.0000	0.8550
0.5	ACR	NC	1.0000	1.0000	0.8550
		IC	1.0000	1.0000	0.8950
		AC	1.0000	1.0000	0.8500
	PCR	NC	1.0000	1.0000	0.8650
		IC	1.0000	1.0000	0.9100
		AC	1.0000	1.0000	0.8550

Table 11 shows the comparison of several test assembly methods for the 200 repetitions under the R-RUM. Like the DINA and DINO model, the performance of MMD test assembly method based on SD_*K*−*L*_ distance is better than the other two methods, and the performance is almost the same under the condition of both no constraints and attribute constraints.

**TABLE 11:** Comparison of the accuracy rate of each method for four attributes under R-RUM model.

Correlation	Accuracy	Constraints	Random	CDI	SD_*K*−*L*_
0	ACR	NC	0.8450	0.9386	0.9514
		IC	0.8447	0.9258	0.9311
		AC	0.8453	0.9408	0.9513
	PCR	NC	0.5418	0.7894	0.8272
		IC	0.5446	0.7594	0.7780
		AC	0.5427	0.7984	0.8269
0.5	ACR	NC	0.8798	0.9504	0.9590
		IC	0.8806	0.9416	0.9443
		AC	0.8801	0.9518	0.9589
	PCR	NC	0.6230	0.8223	0.8495
		IC	0.6272	0.7986	0.8081
		AC	0.6240	0.8275	0.8492

Table 12 shows the comparison of the accuracy rate of each method when the number of attributes is four across replications under the R-RUM. It can be seen from Table 12 that in the 200 simulation replications, the MMD test assembly method based on SD_*K*−*L*_ distance is better than the CDI test assembly method in every case, and its performance on R-RUM is also better than that of DINO model under the condition of both no constraints and attribute constraints.

**TABLE 12:** Comparison of the accuracy rate of each method for four attributes under the R-RUM model.

Correlation	Accuracy	Constraints	CDI Outperforms Random	SD_*K*−*L*_ Outperforms Random	SD_*K*−*L*_ Outperforms CDI
0	ACR	NC	1.0000	1.0000	0.9750
		IC	1.0000	1.0000	0.7400
		AC	1.0000	1.0000	0.9700
	PCR	NC	0.9950	1.0000	0.9650
		IC	0.9950	1.0000	0.7850
		AC	1.0000	1.0000	0.9550
0.5	ACR	NC	1.0000	1.0000	0.9650
		IC	1.0000	1.0000	0.7050
		AC	1.0000	1.0000	0.9700
	PCR	NC	1.0000	1.0000	0.9600
		IC	1.0000	1.0000	0.7300
		AC	1.0000	1.0000	0.9650

Tables 13–18 show accuracy rates and comparison results for eight attributes. The results of eight attributes are similar to that of four attributes. On the whole, the new method is better than the CDI test assembly method and the random assembly method under the DINA model, the DINO model and the R-RUM. Furthermore, the new method has a greater advantage over the CDI method in terms of the PCR. Under the three models, the PCR of the MMD test assembly method based on SD_*K*−*L*_ distance is higher than that of the CDI test assembly method, but the ACR of the MMD test assembly method is slightly lower than the CDI test assembly method. It means that the higher the averaged ACR, the PCR is not necessarily higher. For example, the ACRs for two attributes are 0.1 and 0.9 or 0.4 and 0.4. Although the average of ACR for these two cases are 0.5 and 0.4, the former case has the PCR of 0.09, while the latter case has the PCR of 0.16, if the correct classification rates for two attributes are independent.

**TABLE 13:** The accuracy rate of each condition for eight attributes under the DINA model.

Correlation	Accuracy	Constraints	Random	CDI	SD_*K*−*L*_
0	ACR	NC	0.6234	0.8294	0.8181
		IC	0.6489	0.7244	0.7289
		AC	0.6234	0.8315	0.8181
	PCR	NC	0.0988	0.3305	0.3678
		IC	0.1304	0.2525	0.2764
		AC	0.0988	0.3438	0.3678
0.5	ACR	NC	0.7474	0.8745	0.8664
		IC	0.7672	0.8267	0.8259
		AC	0.7474	0.8759	0.8664
	PCR	NC	0.3272	0.4683	0.4909
		IC	0.3381	0.4479	0.4547
		AC	0.3272	0.4766	0.4909

**TABLE 14:** Comparison of the accuracy rate of each method for eight attributes under the DINA model.

Correlation	Accuracy	Constraints	CDI Outperforms Random	SD_*K*−*L*_ Outperforms Random	SD_*K*−*L*_ Outperforms CDI
0	ACR	NC	1.0000	1.0000	0.1500
		IC	1.0000	1.0000	0.6950
		AC	1.0000	1.0000	0.0950
	PCR	NC	1.0000	1.0000	0.8850
		IC	1.0000	1.0000	0.9600
		AC	1.0000	1.0000	0.8350
0.5	ACR	NC	1.0000	1.0000	0.1000
		IC	1.0000	1.0000	0.4150
		AC	1.0000	1.0000	0.0650
	PCR	NC	1.0000	1.0000	0.9400
		IC	1.0000	1.0000	0.7800
		AC	1.0000	1.0000	0.8900

**TABLE 15:** The accuracy rate of each condition for eight attributes under the DINO model.

Correlation	Accuracy	Constraints	Random	CDI	SD_*K*−*L*_
0	ACR	NC	0.6208	0.8289	0.8171
		IC	0.6479	0.7238	0.7296
		AC	0.6208	0.8302	0.8171
	PCR	NC	0.0963	0.3319	0.3679
		IC	0.1294	0.2525	0.2781
		AC	0.0963	0.3435	0.3679
0.5	ACR	NC	0.7444	0.8754	0.8665
		IC	0.7666	0.8268	0.8264
		AC	0.7444	0.8765	0.8665
	PCR	NC	0.3248	0.4706	0.4910
		IC	0.3368	0.4488	0.4553
		AC	0.3248	0.4776	0.4910

**TABLE 16:** Comparison of the accuracy rate of each method for eight attributes under the DINO model.

Correlation	Accuracy	Constraints	CDI Outperforms Random	SD_*K*−*L*_ Outperforms Random	SD_*K*−*L*_ Outperforms CDI
0	ACR	NC	1.0000	1.0000	0.1900
		IC	1.0000	1.0000	0.7200
		AC	1.0000	1.0000	0.1600
	PCR	NC	1.0000	1.0000	0.8750
		IC	1.0000	1.0000	0.9400
		AC	1.0000	1.0000	0.8450
0.5	ACR	NC	1.0000	1.0000	0.0700
		IC	1.0000	1.0000	0.4350
		AC	1.0000	1.0000	0.0400
	PCR	NC	1.0000	1.0000	0.8950
		IC	1.0000	1.0000	0.8000
		AC	1.0000	1.0000	0.8700

**TABLE 17:** The accuracy rate of each condition for eight attributes under the R-RUM model.

Correlation	Accuracy	Constraints	Random	CDI	SD_*K*−*L*_
0	ACR	NC	0.7427	0.8300	0.8336
		IC	0.7572	0.8142	0.8231
		AC	0.7427	0.8303	0.8336
	PCR	NC	0.1236	0.2648	0.2823
		IC	0.1365	0.2433	0.2651
		AC	0.1236	0.2660	0.2823
0.5	ACR	NC	0.8243	0.8753	0.8787
		IC	0.8300	0.8705	0.8745
		AC	0.8243	0.8757	0.8787
	PCR	NC	0.3261	0.4175	0.4309
		IC	0.3232	0.4183	0.4254
		AC	0.3261	0.4189	0.4309

**TABLE 18:** Comparison of the accuracy rate of each method for eight attributes under the R-RUM model.

Correlation	Accuracy	Constraints	CDI Outperforms Random	SD_*K*−*L*_ Outperforms Random	SD_*K*−*L*_ Outperforms CDI
0	ACR	NC	1.0000	1.0000	0.6350
		IC	1.0000	1.0000	0.8700
		AC	1.0000	1.0000	0.6350
	PCR	NC	1.0000	1.0000	0.8000
		IC	1.0000	1.0000	0.8850
		AC	1.0000	1.0000	0.7950
0.5	ACR	NC	1.0000	1.0000	0.7900
		IC	1.0000	1.0000	0.8450
		AC	1.0000	1.0000	0.7800
	PCR	NC	1.0000	1.0000	0.8300
		IC	1.0000	1.0000	0.6850
		AC	1.0000	1.0000	0.8300

## Discussion

Simulation results show that the MMD test assembly method with the simplified constraints has similar performance to the new method with the full constraints under four attributes, and the new simplified method performs better than the CDI method for four and eight attributes in term of the PCR. The MMD test assembly method with the full constraints suffers a large computational burden due to the optimization problem of complex constraints, but it is fast and performs relatively well when the number of attributes is four. In order to simplify computation, the MMD test assembly method with the simplified constraints can simplify computation effectively and is suitable for a larger number of attributes (i.e., eight attributes). We also found that when the number of measured attributes increases, the advantages in PCR for the MMD method are still obvious, while its performance in ACR tends to be average. This is related to the characteristics of the MMD and CDI-based test assembly methods: the CDI test assembly method pays attention to the local information, while the MMD focuses on the global information. When the ratio of test length to attribute number is large, the MMD test assembly method has enough room to play and select enough high-quality tests to obtain sufficient overall information, in order to make up for the lack of local information. So, the MMD test assembly method has obvious advantages at this condition.

We found that there is a considerably worse performance for item constraints compared to attribute constraints, which is consistent of results of Henson and Douglas (2005). The possible explanations are as follows: First, we think this may be related to the concept of statistical identification that is receiving a lot of attention lately for the case of CDMs. Specifically, for the DINA model, two identity matrices in the Q matrix and an additional third item per attribute would be required (e.g., Chen et al., 2015; Xu and Shang, 2018). This would be never satisfied in the item constraint condition. Second, because the item constraints required only 4 items measured one attribute, the Q-matrix is not complete if all columns of the *K* × *K* identity matrix are not contained in the Q-matrix. A simple example of a complete Q-matrix is the *K* × *K* identity matrix ***I*** (Chiu et al., 2009; Cai et al., 2018). Third, item-level expected classification accuracy of attributes for 16 items measured two or three attributes in item constraint condition is often lower than that for items measured only one attribute (Wang et al., 2019).

Test constraints in this study are still rough, since it is only a repetition of Henson and Douglas (2005) experiments. The performance of each method under other constraints needs to be studied. The MMD test assembly method with SD_*K*−*L*_ distance as the index is superior to CDI test assembly method in performance, but the combination of this test assembly idea and other distance indexes is worth discussing. This study does not consider the relationship between the number of measured attributes and the length of tests, the influence of the ratio of the test length and number of attributes on the MMD test assembly method, how about the specific relationship between them is, and how to specify item constraints and attribute constraints when the length of tests is different, all of above will need a further investigation.

As in the study of Henson and Douglas (2005), the larger sample in our study was only employed to obtain correct classification rates more stability with simulated item parameters. We have not considered the impact of item banks calibrated by using larger or smaller sample sizes on the performance of test construction methods. As the reviewer motioned, it is true that larger sample sizes are likely to be used to calibrate item banks (e.g., Liu et al., 2013; George and Robitzsch, 2014), while the review of available empirical studies indicates that sample sizes in cognitive diagnosis tend to be much smaller (Sessoms and Henson, 2018). It would be an interesting question to justify whether a difference in performance is expected from the CDI and SD_*K*−*L*_ methods for item banks calibrated from different sample sizes. One limitation of this study is that three simple CDMs (the DINA model, the DINO model, and the R-RUM) were considered in the simulation study. If we have a large sample size to calibrate an item bank, we believe that the results can be generalized to a more general model, such as the G-DINA model (de la Torre, 2011), or a combination of reduced models (Ravand, 2016; Sorrel et al., 2017; de la Torre et al., 2018).

## Data Availability Statement

The raw data supporting the conclusions of this article will be made available by the authors, without undue reservation.

## Author Contributions

WW and LS designed the study and wrote the manuscript. JZ, YT, and PG drafted and revised the manuscript. WW and PG conducted the simulation study. All authors contributed to the article and approved the submitted version.

## Conflict of Interest

The authors declare that the research was conducted in the absence of any commercial or financial relationships that could be construed as a potential conflict of interest.
